# Application of a replicative targetable vector system for difficult-to-manipulate streptomycetes

**DOI:** 10.1007/s00253-025-13477-3

**Published:** 2025-04-10

**Authors:** Juan Pablo Gomez-Escribano, Alina Zimmermann, Shu-Ning Xia, Meike Döppner, Julia Moschny, Chambers C. Hughes, Yvonne Mast

**Affiliations:** 1 Department Bioresources for Bioeconomy and Health Research, Leibniz Institute DSMZ-German Collection of Microorganisms and Cell Cultures, Inhoffenstraße 7B, 38124 Braunschweig, Germany; 2 German Center for Infection Research (DZIF), Partner Site Tübingen, 72076 Tübingen, Germany; 3 Department of Microbial Bioactive Compounds, Interfaculty Institute of Microbiology and Infection Medicine (IMIT), University of Tübingen, Auf der Morgenstelle 28, 72076 Tübingen, Germany; 4 Cluster of Excellence EXC 2124: Controlling Microbes to Fight Infection, University of Tübingen, 72076 Tübingen, Germany; 5 Braunschweig Integrated Centre of Systems Biology (BRICS), Rebenring 56, 38106 Braunschweig, Germany; 6 Institute for Microbiology, Technical University Braunschweig, Rebenring 56, 38106 Braunschweig, Germany

**Keywords:** *Streptomyces*, Genetic manipulation, Homologous recombination, Knock-out, Genetic tools

## Abstract

**Abstract:**

The low frequency of homologous recombination together with poor efficiency in introducing DNA into the cell are the main factors hampering genetic manipulation of some bacterial strains. We faced this problem when trying to construct mutants of *Streptomyces iranensis* DSM 41954, a strain in which conjugation is particularly inefficient, and suicidal vectors had failed to yield any exconjugants. In this work, we report the construction and application of a conjugative replicative vector, pDS0007, which allows selection of exconjugants even with poor conjugation efficiency. The persistence of the construct inside the cell for as long as required facilitates the homologous recombination events leading to single and double crossovers. While it was confirmed that the vector is frequently lost without selection, the recognition sequence for the I-SceI endonuclease was included in the backbone of pDS0007. The presence of a I-SceI recognition sequence would allow to force the loss of the vector and the appearance of double crossover recombinants by introducing a second construct (e.g. pIJ12742) that expresses a *Streptomyces* codon–optimised gene encoding the I-SceI endonuclease. To facilitate screening for vector-free clones, the construct also carries a *Streptomyces* codon–optimised *gusA* gene encoding the β-glucuronidase expressed from a constitutive promoter. We prove the usefulness of this vector and strategy with the strain *S. iranensis* DSM 41954, in which we could readily delete an essential gene of a newly discovered biosynthetic pathway for a phosphonate-containing natural product, which led to loss of phosphonate production according to ^31^P NMR spectroscopy.

**Key points:**

*• pDS0007 is a new vector for gene-targeting in difficult-to-manipulate streptomycetes.*

*• pDS0007 is self-replicative but easy to cure, targetable and allows visual screening.*

*• pDS0007 was used to prove the discovery of a novel phosphonate biosynthetic pathway.*

**Graphical Abstract:**

**Figure:**
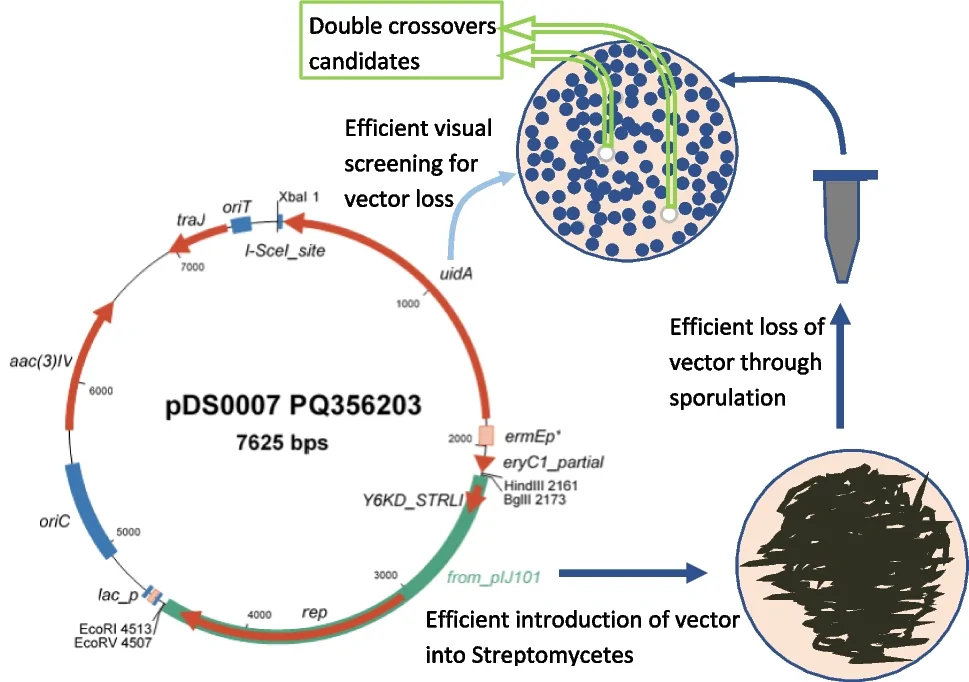

**Supplementary Information:**

The online version contains supplementary material available at 10.1007/s00253-025-13477-3.

## Introduction

Streptomycetes and closely related filamentous actinomycetes are high mol % G + C Gram-positive bacteria that have gained the attention of molecular microbiologists for over half a century; first because of their very complex life cycle, resembling that of filamentous fungi, and mostly because of their unmatched capacity to produce bioactive natural products of outstanding importance for medicine, particularly antimicrobials (Katz and Baltz 2016).

Genetics studies based on recombinant DNA technologies have been extensively applied to the study of the biology of streptomycetes and specialised metabolism (Kieser et al. 2000; Baltz 2006). While novel technologies like CRISPR-Cas9 are now available for genetic manipulation in streptomycetes (Alberti and Corre 2019), gene targeting is still mostly based on homologous recombination, wherein a gene is replaced with a marker or mutated allele. To deliver the recombination construct into the *Streptomyces* cell, non-replicative (suicidal) vectors are the first choice to facilitate selection of at least single crossovers before proceeding to segregation and selection of double crossover mutants (Kieser et al. 2000).

Non-replicative vectors are however unsuitable for strains in which DNA introduction or homologous recombination is not efficient, which is a requirement for stabilising the construct in the genome by a first crossover event before the non-replicative construct is lost. Temperature-sensitive replicative vectors are then the choice, to facilitate the selection of transformants (or exconjugants) without the requirement of homologous recombination to maintain the construct. The stable presence of the construct inside the cell facilitates the required homologous recombination crossover events, while being able to counter-select non-recombinants by cultivation at the non-permissive higher temperature (Muth 2018). These vectors are however very stable and cultivation at the required temperature to abolish replication has been suggested to be mutagenic (Mo et al. 2019), if possible at all for many streptomycetes.

One possibility to address this problem is to employ vectors that are replicative but can be easily cured, either due to intrinsic instability or that carry genetic elements that facilitate forcing the loss of the vector. pIJ101 is a natural plasmid isolated from *Streptomyces lividans* ISP 5434 (Kieser et al. 1982) whose replication functionality has been extensively used in multiple vectors for genetic manipulation in *Streptomyces* (Kieser et al. 2000). pIJ101-*ori*-*rep*-based vectors have been constructed mostly for ectopic gene expression but also for cloning and gene replacement (Kieser et al. 1982; Kendall and Cohen 1988; Brasch and Cohen 1995). Particularly useful for gene replacement are pIJ101-derived vectors with decreased segregational stability (Sun et al. 2009). pIJ86 is an *Escherichia coli–Streptomyces* bifunctional conjugatable vector constructed by Helen Kieser (John Innes Centre, Norwich, UK; Mervyn Bibb, personal communication) as a gene expression vector with the constitutive promoter of the erythromycin resistance gene (ermE*p), and has been successfully used as such for protein production in *Streptomyces* (Berini et al. 2020). pIJ86 can be mobilised to streptomycetes by RK2/RP4 conjugation from *E. coli.* pIJ86 relies on the origin of replication (*ori*) and replication protein (*rep*) gene from plasmid pIJ101 for replication in *Streptomyces* and it carries the selectable marker *aac(3)IV* for apramycin resistance (for both, *Streptomyces* and *E. coli*). Although it was designed for gene expression and therefore with stability in mind, pIJ86 has been used for CRISPR-Cas9-based genome editing and shown to be easily lost if antibiotic selection is not maintained (Gomez-Escribano et al. 2021).

During a project focused on the identification of novel strains producers of phosphonate-containing natural products (Zimmermann et al. 2023, 2024), we encountered the need for genetic manipulation of *Streptomyces iranensis* DSM 41954 (original designation *S. iranensis* HM 35). This is a biotechnologically interesting strain as it is an alternative rapamycin producer and with promising potential as source for novel bioactive natural products (Horn et al. 2014). However, genetic manipulation of this strain is particularly challenging because introduction of DNA is inefficient (Netzker et al. 2016). To address this, we developed a vector that would enable to create mutants in this strain, and other streptomycetes that are difficult to genetically manipulate.

In this study, we describe the construction of a gene-targeting vector based on pIJ86. pDS0007 vector includes the *gusA* gene encoding the β-glucuronidase for visual screening of plasmid presence. It also carries the recognition sequence for the endonuclease I-SceI to force the loss of the vector when not lost through segregation, and, more importantly, to force the DNA double-strand break repair by homologous recombination, thereby facilitating the appearance of gene-replacement mutants (Siegl et al. 2010; Fernández-Martínez and Bibb 2014). We prove that the vector is quickly lost without antibiotic selection despite being self-replicative, and investigate possible genetic causes of this rapid curing. We further test its usability and advantages by constructing a gene-replacement mutant in the phosphonate natural compound producer *S. iranensis* DSM 41954.

## Materials and methods

### Bacterial strains, plasmids and cultivation conditions

Plasmids and strains used or generated during this study are listed in Supplementary Tables S1 and S2 respectively.

pIJ86 (aac(3)IV, RK2-oriT, ColE1-ori, ermE*p*, pIJ101-ori-rep*) and pSET152 (Bierman et al. 1992) (*aac(3)IV, RK2-oriT, ColE1-ori, phiC31-int, phiC31-attP*) and their DNA sequences were a gift from JIC StrepStrains (jic.strepstrains@jic.ac.uk, John Innes Centre, Norwich Research Park, Norwich, NR4 7UH, UK). pGM1190 (Muth 2018) (*tsr*, *aac(3)IV*, *oriT*, *to* terminator *tipA*p, RBS, *fd* terminator, *sso*, *rep*_ts_) and pGus21 (aac(3)IV, oriT, ermE*p*_gusA, oripMB1*; knock-out vector, non-replicative in *Streptomyces*) and their DNA sequence were a gift from Günther Muth (Sigle et al. 2016) (Eberhard Karls Universität Tübingen, Geschwister-Scholl-Platz, 72,074 Tübingen, Germany). Restriction endonucleases, T4-DNA ligase, Q5 and One-Taq DNA polymerases, DNA Polymerase I-Large (Klenow) Fragment, were purchased from New England Biolabs (New England Biolabs GmbH, Brüningstraße 50; Geb. B852 (Industriepark Höchst), D- 65926 Frankfurt am Main, Germany) and used according to provider’s instructions.

*Streptomyces coelicolor* strains M145 (Kieser et al. 2000) and M512 (Floriano and Bibb 1996), and *Escherichia coli* ET12567/pUZ8002 (MacNeil et al. 1992; Paget et al. 1999) were a gift from JIC StrepStrains; *Streptomyces iranensis* DSM 41954 (Hamedi et al. 2010) was sourced from the German Collection of Microorganisms and Cell Cultures (Leibniz-Institut DSMZ-Deutsche Sammlung von Mikroorganismen und Zellkulturen GmbH, Inhoffenstraße 7 B, 38,124 Braunschweig, Germany); *E. coli* DH5α (Bethesda Research Laboratories 1986; Grant et al. 1990) was purchased from New England Biolabs (as NEB5-alpha) or sourced from DSMZ collection (strain DSM 6897).

*E. coli* cultivation and general molecular biology techniques were performed following established methods (Sambrook et al. 1989) and instructions provided by suppliers. *Streptomyces* strains were cultivated in SFM (MS) for preparation of spore stocks, and for mobilisation of plasmid constructs to *Streptomyces* strains by conjugation from *E. coli*, which were done according to established methods (Kieser et al. 2000) or with the modifications by Netzker and co-workers (Netzker et al. 2016) when indicated for *S. iranensis*. Apramycin and kanamycin were used at 50 mg/L, fosfomycin at 20 mg/L, final concentration for both *E. coli* and *Streptomyces*. For the comparative conjugation efficiency studies, the same culture of donor *E. coli* was split into two equal fractions and mixed with 0.5 mL of pregerminated spores of either *S. coelicolor* or *S. iranensis*; efficiency was calculated as number of colonies that appeared on the conjugation plates (full accurate count for *S. iranensis* as the number was very low, estimation for *S. coelicolor* as the number was very high) divided by the estimated number of viable spores used.

Genomic DNA was purified following the salting-out method (Kieser et al. 2000) from mycelium of *S. iranensis* cultivated in liquid S medium (10 g glucose, 10 g glycerol, 4 g peptone, 4 g yeast extract, 4 g K_2_HPO_4_, 2 g KH_2_PO_4_, 0.5 g MgSO_4_ dissolved in 1 L distilled water) in baffled flasks and orbital shaking (160 rpm) at 28 °C for 7 days.

### DNA sequencing and sequence analysis

DNA was sequenced using the Sanger sequencing (Sanger et al. 1977) and Oxford Nanopore Technologies-ONT (Oxford Nanopore Technologies plc. Gosling Building, Edmund Halley Road, Oxford Science Park, OX4 4DQ, UK). Whole Plasmid Sequencing services provided by Eurofins Genomics (Eurofins Genomics Europe Shared Services GmbH; Anzinger Str. 7a; 85,560 Ebersberg; Germany). Informatic DNA sequence analysis was performed with Clone Manager 11 (Sci Ed Software LLC, Westminster, Colorado, USA), ApE-A plasmid Editor v3.0.8 (Davis and Jorgensen 2022), Artemis release 17.0.1 (Rutherford et al. 2000) and Staden Package version 2.0.0b11 - 2016 (Staden et al. 1999; Bonfield and Whitwham 2010). Annotation of plasmid genetic elements was done with pLannotate (McGuffie and Barrick 2021). Search and annotation of putative biosynthetic gene clusters were performed with antiSMASH version 7 (Blin et al. 2023). Homology search was performed at NCBI BLAST server (Altschul et al. 1997) and locally with prfectBLAST (Santiago-Sotelo and Ramirez-Prado 2012).

pIJ86 de novo sequence was first obtained by Sanger sequencing; reads obtained with oligonucleotides in Supplementary Table S3 were assembled with GAP4 of Staden Package (Staden et al. 1999; Bonfield and Whitwham 2010) to generate a consensus. Independently, pIJ86 was sequenced with Oxford Nanopore Technologies (Eurofins Genomics) (which with a coverage of 854 × provided a reliable sequence that fully matched the manually curated Sanger assembly). The ONT sequence was annotated on first instance with pLannotate (McGuffie and Barrick 2021) and then manually curated. The KpnI site, most likely used for the cloning of ermE*p, was used as the starting position for the circular sequence. The final annotated sequence was deposited at NCBI under accession number PQ361717.

### β-glucuronidase assay

Detection of β-glucuronidase activity was performed based on the previously published methods (Myronovskyi et al. 2011) with modifications: X-Gluc, 5-Bromo- 4-chloro- 3-indolyl-β-D-glucuronide, was purchased as powder (Carl Roth GmbH + Co. KG, catalogue number 0018.1; AppliChem GmbH catalogue number A1113,0001) and dissolved in N,N′-dimethylformamide to 40 mg/mL stock solution, prepared just before using it (due to the short shelf-life of the stock, even at − 20 °C); X-Gluc was added to melted SFM medium at 55 °C at a final concentration of 40 mg/L.

### Molecular cloning

Oligonucleotides used during this study are listed in Supplementary Table S3.

#### Construction of pDS0201

Two homologous DNA fragments of about two kilobases (kb) flanking *S. iranensis* DSM 41954 *pepM* (which lays between positions 128,908 and 129,582 of sequence with accession JAGGLR010000028.1) were PCR amplified with oligonucleotides AZ029-AZ030 (downstream) and AZ031-AZ033 (upstream) (Table S1) and Q5 high-fidelity DNA polymerase (New England Biolabs). Each PCR amplicon was first cloned as a blunt-ended DNA fragment in pBluescript II KS + linearized with SmaI, and their correctness and directionality in the cloning vector were assessed by Sanger sequencing with universal primers M13 F- 24 mer and M13R- 22 mer (Table S1). The upstream fragment was excised with EcoRI-HindIII (sites present in primers) and ligated to pGus21 linearized with the same enzymes. The kanamycin resistance gene *neo* was excised from pTC192-Km (Rodríguez-García et al. 2006) with XbaI and ligated to pBluescript-downstream fragment linearized with XbaI, and the desired orientation selected. The downstream-*neo* insert was then excised with HindIII and ligated to pGus21-upstream linearized with HindIII, and the correct orientation was selected. This resulted in the construct pDS0201.

#### Construction of pDS0202

The homologous recombination cassette was excised from pDS0201 as a 4.8 kb NruI fragment (sites for NruI were located at the edge of the homologous fragments) and ligated to pDS0007 linearized with EcoRV, yielding pDS0202.

#### Construction of pDS0204

A DNA fragment containing *S. iranensis pepM* was PCR amplified with oligonucleotides AZ072-AZ073 and cloned into pBluescript II KS + linearized with SmaI. This construct was used as template for a nested PCR with oligonucleotides AZ078-AZ079, which carry recognition sequence for NdeI and HindIII restriction enzymes; Q5 high-fidelity DNA polymerase (New England Biolabs) was used for all PCR reactions. The AZ078-AZ079 PCR product was then digested with these enzymes and ligated to pRM4 linearized with the same enzymes, leading to plasmid pDS0204. Selected clones were confirmed by Sanger sequencing with universal primers M13 F- 24 mer and M13R- 22 mer.

#### Construction of pDS0007

pIJ86 was cut with HindIII, ends refilled and cut with XbaI. pGus21 (Sigle et al. 2016) was cut with XhoI, ends refilled with DNA Polymerase I-Large (Klenow) Fragment and cut with XbaI; the blunt-XbaI DNA segment carrying ermE*p/RBS/*gusA*/I-SceI-site was purified from an agarose gel after electrophoresis and ligated to the blunt-XbaI linearized pIJ86 vector to generate pDS0007. One of the clones that showed the expected arrangement on agarose electrophoresis was confirmed by whole plasmid sequencing with Oxford Nanopore Technologies (coverage 676x, Eurofins Genomics), annotated with pLannotate (McGuffie and Barrick 2021) and then manually curated. The unique XbaI site was used as the starting position for the circular sequence. The final annotated sequence of pDS0007 was deposited at NCBI with accession number PQ356203. pDS0007 was deposited at the DSMZ open collection (number DSM 119277).

### ^*31*^*P NMR analysis for phosphonate detection*

*S. iranensis* strains were cultivated in 100-mL Erlenmeyer baffled flasks containing 30 mL of R5 medium (Kieser et al. 2000), with orbital shaking (180 rpm) at 28 °C for 3 to 4 days. These cultures (5 mL) were used to inoculate 30 mL of GUBC medium in 100-mL baffled flasks (GUBC medium: 10 g saccharose, 5 g meat extract, 5 g casamino acids, 5 g glycerol, 5 mL 1 M Na_2_HPO_4_/KH_2_PO_4_ pH 7.3, 2 mL Hunter’s base dissolved in 1 L distilled water, pH adjusted to 7.3 and 10 mL Balch’s vitamins added after autoclaving. Hunter’s concentrated base: 20 g nitrilotriacetic acid, 14 g KOH, 59.3 g MgSO_4_^.^7H_2_O, 6.67 g CaCl_2_^.^2H_2_O, 0.0185 g (NH_4_)_6_Mo_7_O_24_^.^4H_2_O, 0.198 g FeSO_4_^.^7H_2_O, 0.25 g EDTA, 1.095 g ZnSO_4_^.^7H_2_O, 0.5 g FeSO_4_^.^7H_2_O, 0.154 g MnSO_4_^.^H_2_O, 0.0392 g CuSO_4_^.^5H_2_O, 0.025 g Co(NO_3_)_2_^.^7H_2_O, 0.0177 g Na_2_B_4_O_7_^.^10H_2_O dissolved in 1 L distilled water, pH adjusted to 6.8. Balch’s vitamins: 5 mg p-aminobenzoic acid, 2 mg folic acid, 2 mg biotin, 5 mg nicotinic acid, 5 mg calcium pantothenate, 5 mg riboflavin, 5 mg thiamine HCl, 10 mg pyridoxine HCl (B6), 100 µg cyanocobalamin (B12), 5 mg thioctic acid (lipoic acid) dissolved in 1 L distilled water, pH adjusted to 7.0, sterilised by filtration). After 7 days, cultures were harvested by centrifugation at 4000 rcf and 4 °C for 15 min. Supernatants were applied to C18 solid-phase extraction (SPE) cartridges (1 g), and the aqueous flow-through was collected. Aqueous phases were diluted with methanol to an 80% final methanol concentration, filtered and dried using a rotary evaporator. ^31^P NMR spectra were recorded in 20% D_2_O at 243 MHz on a Bruker Avance III HDX 600 MHz spectrometer fitted with a 5-mm Prodigy BBO H&F CryoProbe. Phosphorus chemical shifts are reported in ppm relative to a 10 mM L-phosphinothricin HCl reference (δ_P_ 51.2) using a double-chamber coaxial NMR tube. NMR data were analysed using MestReNova 14.3.0.

## Results

### Construction of pDS0007

Based on previous insights on the facile curing of pIJ86-based plasmids (Gomez-Escribano et al. 2021), we chose this vector as the backbone for our gene-targeting vector. To be able to visually screen colonies for the presence or absence of the vector, we chose to include the gene *gusA* in the backbone, which encodes the β-glucuronidase enzyme optimised for *Streptomyces* codon usage (Myronovskyi et al. 2011), expressed from the constitutive promoter ermE*p (Bibb et al. 1994) and an optimised ribosome binding site (RBS) for *Streptomyces* (Kieser et al. 2000)*.* To be able to force the loss of the vector, and the appearance of double crossovers, by directed double-strand DNA break, we added the recognition sequence for the yeast intron-encoded endonuclease I-SceI (Siegl et al. 2010; Fernández-Martínez and Bibb 2014).

**Fig. 1:**
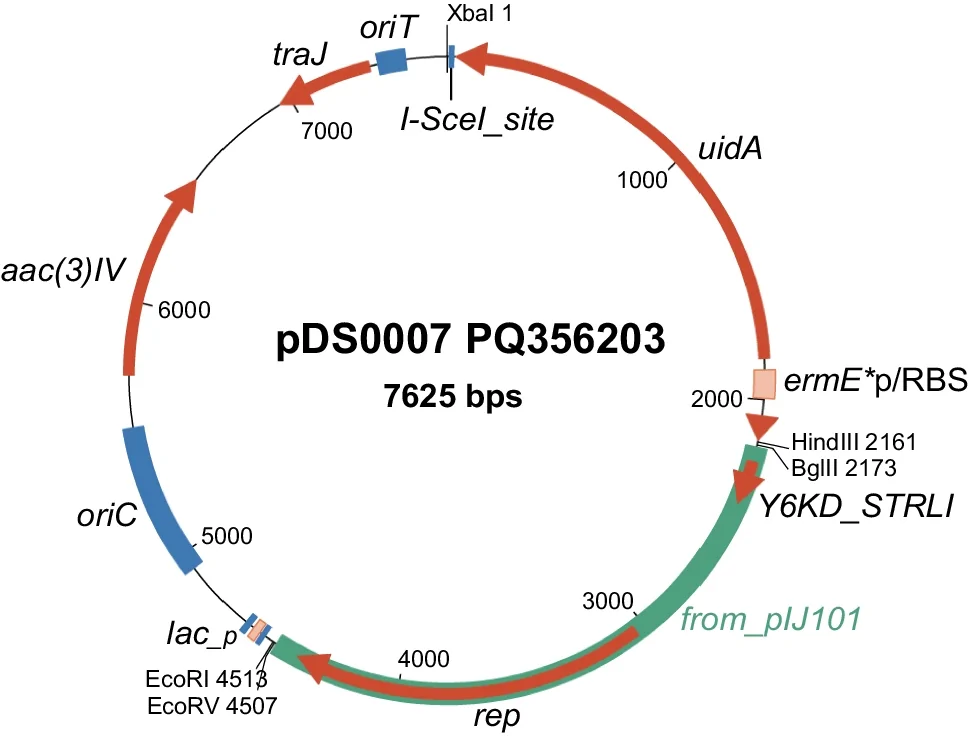
Genetic map of pDS0007, including unique restriction sites for the addition of homologous recombination cassettes or other DNA fragments

The DNA segment carrying ermE*p/RBS/*gusA*/I-SceI-site was excised from pGus21 (Sigle et al. 2016) and ligated to pIJ86 (substituting the original ermE*p promoter from pIJ86) to generate vector pDS0007 (Fig. 1; see “Materials and methods” for details). While there is no remaining multi-cloning site, the deletion cassette can be cloned into several unique restriction sites that are not expected to interfere with plasmid functions: XbaI (used during the construction of pDS0007), HindIII (regenerated at the XhoI-HindIII scar), BglII, EcoRI, NruI or EcoRV (which we used in this study). A full de novo sequence was obtained (see “Materials and methods” and below) and deposited at NCBI with accession number PQ356203. The plasmid is available from the DSMZ open collection with number DSM 119277.

### Assessment of the replication region of pDS0007 derived from pIJ101

An alignment of the pIJ86 sequence (in silico, provided by JIC StrepStrains; Fig. S1) and pHZ1358 sequence (another pIJ101-derived vector, Genbank accession AY667410; Fig. S2) showed that the pIJ101 (Fig. S3) replication region present in pIJ86 is missing the *sti* locus (4366 to 4441 bp in pHZ1358 annotated sequence). A detailed analysis is given in Supplementary Information (Figs. S4 and S5). However, the available pIJ86 sequence was only an in silico compiled sequence with no further information, making it essential to obtain a de novo sequence of the full pIJ86 vector, which we obtained with both Sanger sequencing and ONT independently (see “Materials and methods”); we also obtained a de novo sequence of the replication region directly from pDS0007 (between position 2684 and 6814 bp of the NCBI PQ356203 sequence) by Sanger sequencing, and a whole plasmid sequence with Oxford Nanopore (see “Materials and methods”). Alignment of these sequences with the in silico sequence of pIJ86, and deposited ones of pHZ1358 (Genbank accession AY667410) and pIJ101 (Genbank accession M21778) revealed that the in silico sequence of pIJ86 is very accurate, and we confirmed that the pIJ101 replication region present in pIJ86 and pDS0007 lacks both the *sti* region and the entire sequence between *rep* and *sti* (Figs. S6 to S10; see Supplementary Information for full details). pIJ86 was known to lack the BamHI site present in the wild-type pIJ101 *rep*, but the actual sequence was unknown (filled with “Ns” in the original sequence). Analysis of the pIJ86 de novo sequence showed that the former BamHI site has the same sequence as in pH1358, which, along with the other single nucleotide mismatches identified (see Figs. S7 to S10), indicates that the pIJ101 *rep* region in pIJ86 and pHZ1358 was derived from the same or a closely related predecessor plasmid (one of those previously reported as a having lost the BamHI site by spontaneous mutation Kieser et al. 1982; Sun et al. 2009)).

### Test of conjugation efficiency of pDS0007 and other vectors

Efficiency of plasmid vector mobilisation by conjugation to *S. iranensis* DSM 41954 has been described to be very low (Netzker et al. 2016). We were also unsuccessful obtaining any exconjugants with pDS0201 (described in “Materials and methods”, discussed below), a suicidal vector carrying two homologous fragments, each two kilobases long, regardless of the modifications to the conjugation protocol we applied, including the previously published protocol (Netzker et al. 2016). To test the conjugation efficiency of the newly generated pIJ86-derivative vector pDS0007, we undertook a comparative study with the model strain *S. coelicolor* M145. As control vectors, we used the phiC31-integrative vector pSET152 and the self-replicative vector pGM1190 (Muth 2018) based on the temperature-sensitive *Streptomyces* origin of replication pSG5 used previously in *S. iranensis* (Netzker et al. 2016)*.* All three vectors are *E. coli*–*Streptomyces* bifunctional, conjugatable and selected for with apramycin in both *E. coli* and *Streptomyces*. Exconjugants were obtained from both strains with all three vectors, but while the conjugation plates were completely covered with exconjugants in the case of *S. coelicolor* M145 for all vectors (estimated efficiency > 6 × 10^−4^ exconjugants per spore), an efficiency of only 8.6 × 10^−7^ (pDS0007), 2.6 × 10^−7^ (pSET152) and 1.6 × 10^−6^ (pGM1190) exconjugants per spore was obtained for *S. iranensis*, i.e. between at least two and three orders of magnitude lower efficiency. The final conjugation protocol used was the standard developed for *S. coelicolor* (Kieser et al. 2000), since employing previously published modifications (Netzker et al. 2016) did not appear to have a significant and reproducible effect.

### Test of stability and segregation properties of pDS0007

**Fig. 2:**
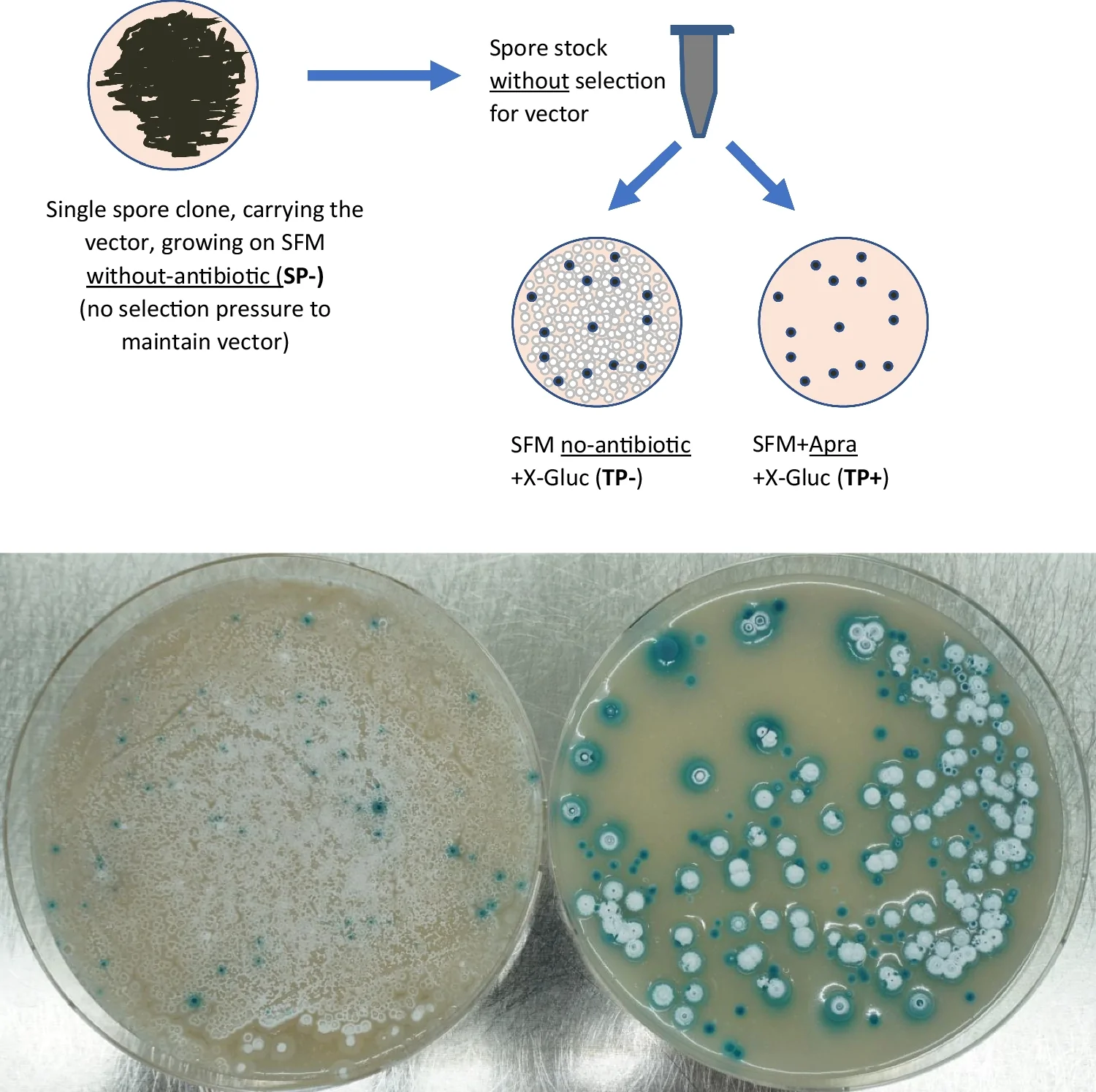
Segregation stability test of pDS0007 in *S. iranensis* DSM 41954. The same volume of a spore preparation obtained from a plate without apramycin selection (i.e. an **SP-** plate in Fig. S11) was used to inoculate two SFM plates supplemented with either only X-Gluc (**TP-**, left) or X-Gluc and apramycin (**TP + **, right). The plate at the left shows confluent growth, indicative of a very high number of colony-forming units inoculated, but only some show conversion of X-Gluc to blue pigment, indicating the very low proportion of spores that have maintained the plasmid. The plate at the right only allows the growth of spores that maintain the plasmid (apramycin selection) and shows a number of individual colonies all of them showing conversion of X-Gluc to blue pigment

The aforementioned observed frequent loss of pIJ86-derived vectors (Gomez-Escribano et al. 2021) was tested in the model strain *S. coelicolor* M145 and in *S. iranensis* DSM 41954. The experimental design to test whether the newly generated pIJ86-derivative vector pDS0007 was stably maintained or readily lost without antibiotic selection, and in comparison to pGM1190, is depicted in Supplementary Information Fig. S11. Exconjugants were replicated on SFM with antibiotic selection for the vector (apramycin), followed by one sporulation round on SFM, either with apramycin or without, as to force the maintenance of the vector or to allow for its loss, respectively. After preparation of spore stocks from each condition, the same volume of a diluted spore stock was plated on SFM with or without apramycin. Colonies developed on each plate were counted after reaching about 2 mm diameter (i.e. allowing sufficient time for all possible colonies to appear). The results showed that when selecting with antibiotic, between 70 and 87% of the spores maintained the plasmids; when spores were developed without antibiotic selection, only 25–28% of spores maintained pGM1190 (with cultivation at the permissive temperature of 30 °C); with pIJ86-derived pDS0007, the plasmid was almost completely lost in *S. coelicolor* and *S. iranensis* (Figs. S12 to S15). Remarkably, in *S. coelicolor*, the vector was almost totally lost as the colonies developed before replica-plating (Fig. S13). An independent experiment to test plasmid loss during growth was a β-glucuronidase assay to visually discriminate colonies originating from spores that have maintained the plasmid (blue pigment production) versus colonies from spores that had lost the plasmid. This allowed screening on the same plate and therefore under exactly the same conditions. The results showed that pDS0007 was lost almost completely after just one round of sporulation without selection, in both *S. coelicolor* M145 and M512 (Figs. S12 to S14), as well as *S. iranensis* (Figs. 2 and S15). Thus, growing under antibiotic, selection forces the maintenance of the vector in most of the spores. It is worth noting that, in both experimental strategies, we were comparing the same spore preparations under either condition and calculating ratios, rather than absolute numbers, of colony count. Therefore, we do not expect our results being influenced by differences of plating efficiency between spore preparations or strains, and the results are a genuine representation of the poor segregational stability of pDS0007.

### Construction of *S. iranensis *Δ*pepM::neo* by using pDS0007 for mutagenesis

**Fig. 3:**
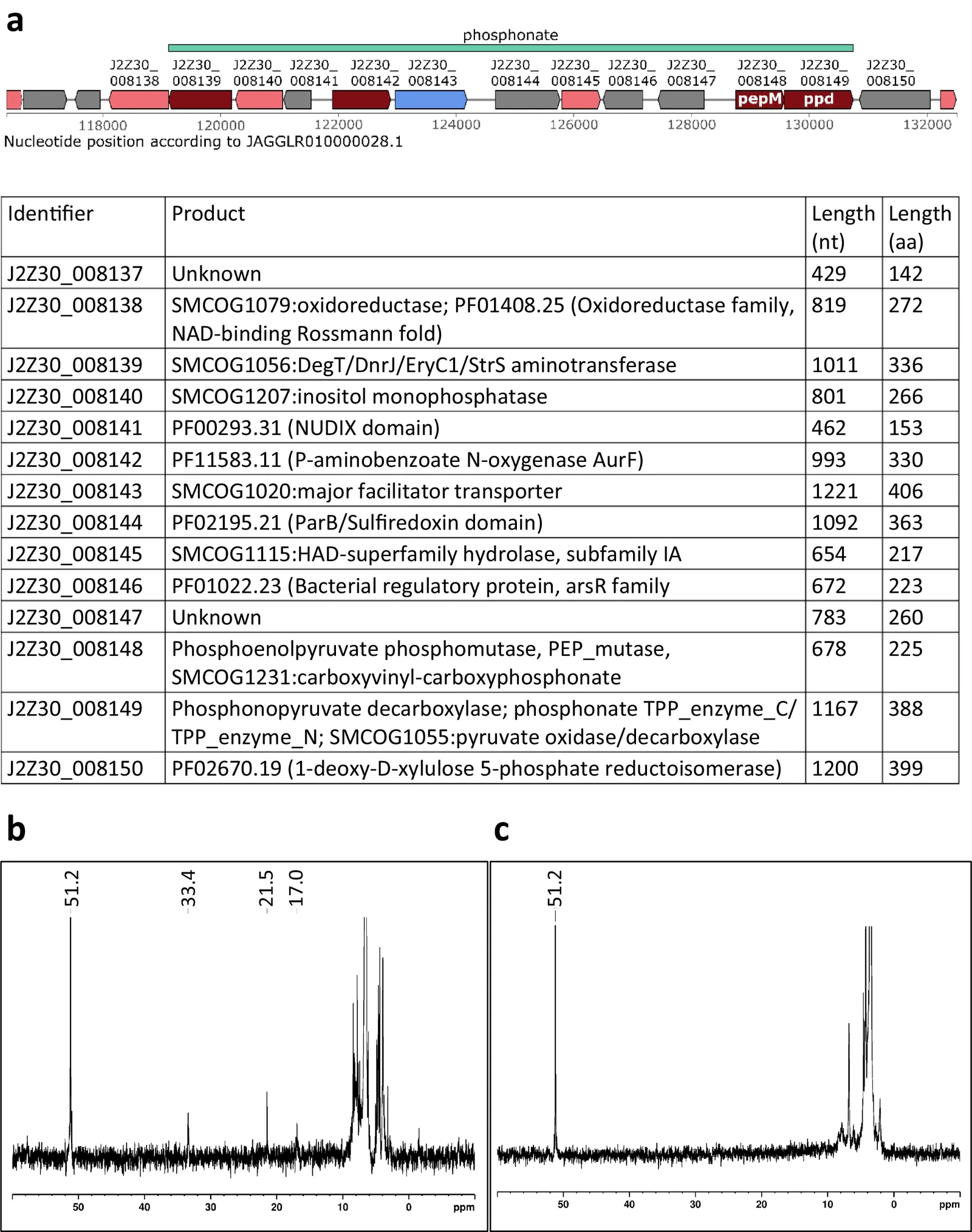
Phosphonate BGC and production of *S. iranensis* DSM 41954. **a** Phosphonate BGC as annotated by antiSMASH on sequence with accession JAGGLR010000028.1. **b** ^31^P NMR spectrum of *S. iranensis* DSM 41954 wild-type showing production of phosphonate-containing metabolites (δ_P_ 33.4, 21.5 and 17.0). **c** ^31^P NMR spectrum of *S. iranensis* Δ*pepM::neo* mutant showing that production of phosphonate-containing metabolites has been abolished. L-phosphinothricin HCl (10 mM, δ_P_ 51.2) was used as a chemical shift reference

As part of a study to screen for new phosphonate antibiotic producers (Zimmermann et al. 2024), *S. iranensis* has recently been identified as a potential phosphonate producer due to the presence of a *pepM* gene in the available draft genome sequence (Horn et al. 2014). *pepM* encodes the enzyme phosphoenolpyruvate mutase (PepM), which is the first and essential enzyme of phosphonate biosynthesis (Kayrouz et al. 2020; Li and Horsman 2022). The putative phosphonate biosynthetic gene cluster is depicted in Fig. 3a. To further study this gene cluster and to uncover its metabolic product, we decided to construct a *pepM* deletion mutant of *S. iranensis* DSM 41954.

For this purpose, a homologous recombination cassette was constructed, with the kanamycin resistance gene *neo* between two homologous DNA fragments of about two kilobases flanking *pepM* (see “Materials and methods” for details). This cassette was first cloned in the suicidal vector pGus21 (construct pDS0201; Fig. S15) and mobilised by conjugation to *S. iranensis* DSM 41954. However, no exconjugants were obtained with this construct under a variety of conditions including the standard ones for *Streptomyces* (Kieser et al. 2000) and those previously published as optimised for *S. iranensis* (Netzker et al. 2016). Thus, we devised the development of pDS0007. The recombination cassette from pDS0201 was transferred to pDS0007 (see “Materials and methods”) and the resulting construct pDS0202 was mobilised to *S. iranensis* DSM 41954. Exconjugants selected for kanamycin resistance were readily obtained with the standard conjugation conditions (Kieser et al. 2000), replicated on SFM supplemented with kanamycin and fosfomycin, and then re-streaked on SFM (without antibiotic selection) for sporulation. The expected phenotype of the mutants is kanamycin resistant, apramycin sensitive and β-glucuronidase negative, due to the integration of the *neo* cassette as a result from homologous recombination by a double crossover event, with simultaneous loss of the vector backbone and thus loss of the *gusA* gene and *apra* cassette. Spores of this “first round” were plated on SFM supplemented with X-Gluc and kanamycin only, allowing growth of both single and double crossover mutants, and enabling screening for presence of vector by the β-glucuronidase-driven conversion of X-Gluc to a blue pigment. Colonies that grew in the presence of kanamycin but did not show blue pigment production were replicated on SFM supplemented with X-Gluc and kanamycin, and then streaked on SFM for sporulation. This “second round” of segregation through sporulation was conducted to ensure that no single crossover or cells with the self-replicating vector were carried over. Spores from the “second round” were plated on SFM supplemented with X-Gluc and kanamycin only. None of the colonies showed blue pigment production nor grew upon replication on SFM with apramycin, clearly indicating they had completely lost the vector backbone, and therefore they were likely double crossovers and candidate mutants. It must be noted that we did not make use of the endonuclease I-SceI to force the loss of vector or second recombination event, since sufficient candidate clones were readily obtained. Ten clones were screened for the expected Δ*pepM::neo* mutant genotype by PCR and all of them showed the expected PCR result (Fig. S16). Two of the candidates were further assessed with additional PCR reactions and Sanger sequencing of the PCR products, and confirmed to have the expected Δ*pepM::neo* genotype (Fig. S17).

### Functional proof of *pepM* deletion in *S. iranensis* by loss of phosphonate production

To prove that the *pepM* gene is involved in phosphonate production in *S. iranensis*, the S*. iranensis* wild-type strain, as well as the Δ*pepM::neo* mutant, was cultivated under phosphonate producing conditions and then analysed for phosphonate production using ^31^P NMR spectroscopy. ^31^P NMR studies revealed putative phosphonate signals (δ_P_ 33.4, δ_P_ 21.5, δ_P_ 17.0) for the *S. iranensis* wild-type samples (Fig. 3b). These signals were absent in the sample of the Δ*pepM::neo* mutant (Fig. 3c) but they were restored upon genetic complementation by ectopic expression of *pepM* from the integrative construct pDS0204 (data not shown). These results show phosphonate production by the *S. iranensis* wild-type strain, which was abolished in the Δ*pepM::neo* mutant, demonstrating that *pepM* is essential for phosphonate production in *S. iranensis* and proving that a *pepM* mutant was successfully generated.

## Discussion

Homology recombination-based genetic manipulation of streptomycetes is often hampered by low efficiency of DNA introduction into the cells combined with low recombinogenicity of the particular strain. Facilitating the persistence of recombination constructs inside the cell increases the chances for homologous recombination. Replicative vectors with temperature-sensitive replication mechanism have been widely adapted as gene-targeting vectors (Bierman et al. 1992; Muth 2018) but it is often not desirable or possible at all to cultivate streptomycetes strains at the non-permissive temperature. In this work, we have constructed a vector that, while being replicative and stably maintained with antibiotic selection, is quickly lost without antibiotic selection, therefore behaving like a suicidal vector.

The vector described in this work, pDS0007, carries the genetic functionality for replication in *E. coli* (colE1 *ori*) and *Streptomyces* (*sti*^−^ pIJ101 *ori*-*rep*), transfer by conjugation from *E. coli* to *Streptomyces* (RK2 = RP4 *ori*), selection in both bacteria with apramycin (*aac(3)IV* marker) and visual screening for vector presence in *Streptomyces* by β-glucuronidase assay (*gusA* = *uidA* marker). The presence of the I-SceI recognition site allows to force the loss of the vector backbone, both when persisting as a self-replicative extrachromosomal plasmid or when integrated as a single crossover, thereby facilitating the appearance of double crossovers. This can be achieved by mobilising a second construct, such as pIJ12742, which expresses a *Streptomyces* codon–optimised gene encoding the I-SceI endonuclease (Fernández-Martínez and Bibb 2014). We have shown that the vector is quickly lost in two *Streptomyces* strains (two derivatives of the model strain *S. coelicolor* A(3)2 and *S. iranensis*) when antibiotic selection is not maintained, and single-spore clones cured from the vector are obtained at very high frequency after just one round of sporulation.

pDS0007 is based on the backbone of pIJ86. The details of the construction process of pIJ86 were never published and only an in silico sequence was available. The thorough analysis of a pIJ86 de novo sequence, together with the information provided by JIC StrepStrains, indicates that pIJ86 is likely a derivative of pSET152 through an intermediate construct named pIJ85, which was also never published (Mervyn Bibb, personal communication). The most likely construction step towards pIJ85 was the ligation of the SacII-SphI fragment of pSET152 (sequence accession AJ414670) that carries the pUC18 *oriC*, the apramycin resistance marker *aac(3)IV* and RP4 conjugation functions (lacking the phiC31 integrative functionality) with a fragment excised from a pIJ101-derived vector that carries the replications functions, including a BamHI-free *rep* gene (the SacII segment between the sites at position 8489 bp and 1970 bp as in pIJ101 sequence with accession M21778). The constitutive ermE*p promoter (Bibb et al. 1994) was then added between the *oriT* and the pIJ101 replication fragment of pIJ85, as a KpnI-HindIII fragment, leading to pIJ86 (as deduced from the sequence information supplied by JIC StrepStrains). It is noticeable that pIJ86 does not contain an optimised ribosome binding site or an NdeI site for cloning of the gene to be expressed. Instead, there is a multi-cloning site with unique sites for BamHI, SphI, HindIII and BglII, that allows the cloning of the gene to be expressed. Another unique restriction site that can be used for cloning additional fragments is the EcoRV site downstream of *rep*, as used in this work for the cloning of the cassette for homologous recombination.

Vectors based on pIJ86 backbone had been previously shown to be segregationally unstable and easily lost without antibiotic selection (Gomez-Escribano et al. 2021) but the reasons remained unknown, and other parts of the construct could have been responsible for the observed high-frequent loss (e.g. CRISPR-Cas9 functionality). During a thorough study of the cloning gene-targeting vector pHZ1358, also based on pIJ101-*ori*-*rep*, Sun and co-workers showed that deletion of the *sti* locus (“site of second strand synthesis “ (Sun et al. 2009) previously called “strong incompatibility locus”(Deng et al. 1988)), located downstream of the replication protein–encoding gene *rep*, results in increased instability and frequent plasmid loss, although its incidence seemed to be strain-dependent (Sun et al. 2009). Our thorough analysis with a newly obtained de novo sequence of pIJ86 shows that pIJ86 lacks the *sti* locus present in pIJ101 and pHZ1358. Sun and co-workers (Sun et al. 2009) referenced previously published work indicating that the lack of *sti* leads to a low plasmid copy number (Deng et al. 1988). The low copy number could be the reason for the quick loss of plasmid from mycelial cellular compartments and, since pIJ86 does not apparently carry any specific plasmid partitioning mechanism that ensures plasmid segregation to spores, the high-frequent number of spores that do not carry the plasmid. It is unlikely that the authors of pIJ86 were aware of the relevance of *sti*, since the pHZ1358 work was published in 2009 (Sun et al. 2009), while pIJ86 was constructed sometime between 2000 (the vector is not included in the Practical Streptomyces Genetics manual (Kieser et al. 2000)) and 2006 (first time we were aware of this vector). Still, it is not certain whether the lack of *sti* and the rest of sequence between *rep* and *sti* is responsible for the observed highly frequent loss of vector, a question that nevertheless goes beyond the aim of this work.

Finally, we have successfully used the vector to create targeted mutants by homologous recombination-based gene replacement in *S. iranensis*, a strain known to be very difficult to manipulate (Netzker et al. 2016). pDS007 was successfully used to delete the gene *pepM* from a newly identified phosphonate biosynthetic gene cluster in *S. iranensis.* The mutants showed the expected genotype and phenotype, with abolished phosphonate production. We show therefore that *S. iranensis* is a natural producer of phosphonate-containing specialised metabolites and that we have correctly identified the responsible biosynthetic gene cluster.

## Supplementary Information

Below is the link to the electronic supplementary material.

Supplementary file1 (DOCX 12407 KB)

## Data Availability

DNA sequences of vectors pIJ86 and pDS0007 were deposited at NCBI under accession numbers PQ361717 and PQ356203 respectively. pDS0007 was deposited at the DSMZ open collection (number DSM 119277).
