# Supplementary material for: Application of a replicative targetable vector system for difficult-to-manipulate streptomycetes
Source: Appl Microbiol Biotechnol. 2025 Apr 10;109:89. doi: 10.1007/s00253-025-13477-3 (PMC11985553; doi:10.1007/s00253-025-13477-3)
Supplement: Supplementary file 1 [file 253_2025_13477_MOESM1_ESM.docx]

**Supporting Information to: “Application of a replicative targetable vector system for difficult-to-manipulate streptomycetes”**

**Table of Contents**

Table S1. Plasmids used in this study	2

Table S2. Strains used in this study	2

Table S3. Oligonucleotides used in this study	3

Assessment of the pIJ101 replication region present in pIJ86 and pDS0007	4

Figure S1. Genetic map of pIJ86	5

Figure S2. Genetic map of pHZ1358	6

Figure S3. Genetic map of pIJ101	7

Figure S4. Pairwise alignment of pIJ86, pHZ1358, and pIJ101	8

Figure S5. Representation of the linearized molecules of pIJ86, pHZ1358, and pIJ101	9

Figure S6. Representation of the GAP4 alignment	10

Figure S7. Analysis of BamHI-free *rep* of pDS0007	11

Figure S8. Assessment of the *rep* region from pIJ86 sequence	12

Figure S9. Comparison of new and *in-silico* pIJ86 sequences	13

Figure S10. Alignment of the pIJ86 sequence reported in this study with JIC’s *in-silico* sequence	14

Figure S11. Study on segregational stability	16

Figure S12. Test of pDS0007 segregation stability	17

Figure S13. Comparative segregation stability test of pGM1190, and pDS0007	18

Figure S14. Segregation stability test of pDS0007 in *S. coelicolor* M512	19

Figure S15. Genetic map of pDS0201	20

Figure S16. Assessment of *S. iranensis* Δ*pepM::neo* candidate mutants by PCR	21

Figure S17. Extended PCR assessment of candidate mutants	22

References	24

Table S1. Plasmids used in this study

| **Name** | **Description** | **Source** |
| --- | --- | --- |
| pIJ86 | *Escherichia coli-Streptomyces* bifunctional expression vector, pIJ101 derived, apramycin resistance *aac(3)IV*, *ermE**p promoter | John Innes Centre, Norwich, United Kingdom |
| pGM1190 | *tipA*p*, tsr, aac(3)IV, rep, sso, dso* | (Muth, 2018) |
| pGus21 | Cloning vector, *aac(3)IV, ermE**p *gusA,* I-SceI-site | (Ladwig *et al.*, 2015) |
| pTC192-km | Source of kanamycin resistance gene *neo* | University of Leon, Spain (Rodríguez-García *et al.*, 2006) |
| pRM4 | *aac(3)IV, ermE**p, *attP_phiC31_, int_phiC31_, oriC_pUC18_, oriT_RP4_* | (Menges *et al.*, 2007) |
| pSET152 | *aac(3)IV,* *attP_phiC31_, int_phiC31_, oriC_pUC18_, oriT_RP4_* | (Bierman *et al.*, 1992) |
| pBluescript II KS(+) | Cloning vector, *bla*, *oriC_pUC18_, lacZ’* | (Alting-Mees and Short, 1989) |
| pDS0007 | pIJ86 derivative with *ermE**p *gusA,* I-SceI-site region from pGus21 | This work |
| pDS0201 | pGus21 derivative with homologous recombination cassette for *pepM* gene replacement | This work |
| pDS0202 | pDS0007 derivative with pDS0201 cassette | This work |
| pDS0204 | pRM4-derivative with *ermE**p::*pepM* | This work |

Table S2. Strains used in this study

| **Name** | **Description** | **Source** |
| --- | --- | --- |
| *Escherichia coli* DH5α | General cloning host  *Δ(argF-lac)169* φ80d*lacZ58*(M15) *ΔphoA8* *glnX44*(AS) *λ^-^* *deoR481 rfbC1* *gyrA96*(NalR) *recA1 endA1 thiE1 hsdR17* | DSMZ; (Bethesda Research Laboratories, 1986; Grant *et al.*, 1990) |
| *Escherichia coli* NEB 5-alpha | General cloning host  *fhuA2Δ(argF-lacZ)U169 phoA glnV44 Φ80Δ(lacZ)M15 gyrA96 recA1 relA1 endA1 thi-1 hsdR17* | New England Biolabs, catalogue number C2987 |
| *Escherichia coli* ET12567/pHZ8002 | Conjugation donor strain  F- *dam*-13::Tn9 *dcm*-6 *hsdM hsdR zjj*-202::Tn10 *recF*143 *galK2 galT22 ara*-14 *lacY*1 *xyl*-5 *leuB6 thi*-1 *tonA*31 *rpsL*136 *his*G4 *tsx*-78 *mtl*-1 *gln* | John Innes Centre, Norwich, United Kingdom; (MacNeil *et al.*, 1992; Paget *et al.*, 1999) |
| *Streptomyces coelicolor* M145 | Prototrophic plasmid free derivative of *S. coelicolor* A3(2) strain | John Innes Centre, Norwich, United Kingdom; (Kieser *et al.*, 2000) |
| *Streptomyces coelicolor* M512 | *S. coelicolor* M145 Δ*actII-ORF4* Δ*redD*  Double deleted mutant derivative of *S. coelicolor* M145 | John Innes Centre, Norwich, United Kingdom; (Floriano and Bibb, 1996) |
| *Streptomyces iranensis* DSM 41954 | Type strain | DSMZ; (Hamedi *et al.*, 2010) |
| *Streptomyces iranensis* YM1001 | DSM 41954 Δ*pepM::neo* | This work |
| *Streptomyces iranensis* YM1003 | DSM 41954 Δ*pepM::neo* *attB_phiC31_*::pDS0204 | This work |

Table S3. Oligonucleotides used in this study

| **Name** | **Sequence** | **Description** |
| --- | --- | --- |
| pIJ10257_Nde_1 | CGAGTGTCCGTTCGAGTG | Sequencing of *ermE**p driven gene |
| pIJ10257_Hind_1 | GTGTTGCCCCAGCAATCAG | Sequencing of *ermE**p driven gene |
| M13F - 24mer | CGCCAGGGTTTTCCCAGTCACGAC | Universal primer for sequencing of insert in many *lacZ*-containing vectors |
| M13R - 22mer | TCACACAGGAAACAGCTATGAC | Universal primer for sequencing of insert in many *lacZ*-containing vectors |
| AZ029 | CTGACCCGTGAAGGGATCTA | Cloning of homologous region downstream of *pepM* |
| AZ030 | CGTGGTACTCAACGGCATTA | Cloning of homologous region downstream of *pepM* |
| AZ031 | CTTAGAATTCGCCCTGCGCCTGATAGGAG | EcoRI site; cloning of homologous region upstream of *pepM* |
| AZ033 | GAATAAGCTTCGCCCAGACGGCTTGGAAC | HindIII site; cloning of homologous region upstream of *pepM* |
| AZ044 | AAGGCCGCTCAACAAGAC | PCR test for *pepM* complementation screening |
| AZ045 | GCGTAGATCACCATGGATACC | PCR test for *pepM* complementation screening |
| AZ066 | TGCTCGACGTTGTCACTG | PCR test for *pepM* mutant screening |
| AZ067 | AGCGGCGATACCGTAAAG | PCR test for *pepM* mutant screening |
| AZ072 | TCGGGAGGAGTACACCTTG | PCR test for *pepM* mutant screening and cloning of *pepM* for complementation |
| AZ073 | CGCCATGCAGAAGTTGTCG | PCR test for *pepM* mutant screening and cloning of *pepM* for complementation |
| AZ074 | TACGGCTGTCGGCATAGAAC | PCR test for *pepM* mutant screening |
| AZ075 | AACTCCGGTGCTTCTCGATG | PCR test for *pepM* mutant screening |
| AZ078 | GGAATCCATATGGCTCATGGCAAACGGGAACC | NdeI site; cloning of *pepM* for complementation |
| AZ079 | CCCAAGCTTCCCGCCATGCAGAAGTTGTC | HindIII site; cloning of *pepM* for complementation |
| AZ084 | GCTCGCCGAGCAAATCTTGG | PCR test for *pepM* mutant screening |
| AZ085 | CCCGAGCAAGATCGTGAGACTG | PCR test for *pepM* mutant screening |
| JP719 | GGAGCGAGTTAGTGCGAAGT | Sanger sequencing of pDS0007 *rep* region |
| JP720 | GTGAGCACCACCACTGTGTC | Sanger sequencing of pDS0007 *rep* region |
| JP721 | CTACCTGCGGCAGATGCT | Sanger sequencing of pDS0007 *rep* region |
| JP722 | CTCGCCAGCCGTCAAGAT | Sanger sequencing of pDS0007 *rep* region |
| JP723 | CGAACACCTTGGGAAAGAAA | Sanger sequencing of pDS0007 *rep* region |
| JP724 | AACTCCTCGGACGGATCG | Sanger sequencing of pDS0007 *rep* region |
| JP725 | CAGGACGACGACCAGGAC | Sanger sequencing of pDS0007 *rep* region |
| JP726 | AGCTCACTCAAAGGCGGTAA | Sanger sequencing of pDS0007 *rep* region |
| JP727 | GGCGGTGCTACAGAGTTCTT | Sanger sequencing of pDS0007 *rep* region |
| JP728 | ACGAGCAACGTTCCTGTCTC | Sanger sequencing of pDS0007 *rep* region |
| JP729 | GTCTCCAGCCGCTTGAAGT | Sanger sequencing of pDS0007 *rep* region |
| JP730 | AGTGAGCGCAACGCAATTA | Sanger sequencing of pDS0007 *rep* region |
| JP731 | CCAAGGTTGAGAAGCTGACC | Sanger sequencing of pDS0007 *rep* region |

Assessment of the pIJ101 replication region present in pIJ86 and pDS0007

To identify the actual replication functions from pIJ101 present in pIJ86 we performed a comparative analysis of the available nucleotide sequences. While the sequence and functional annotation of pIJ101 has been available since 1988 (Kendall and Cohen, 1988), the best studied sequence is that of the pIJ101-derived vector pHZ1358 (Sun *et al.*, 2009) (GenBank AY667410). The published sequence for pIJ101 (GenBank M21778; Figure S1) starts within the replication protein encoding gene *rep*, making it more difficult to obtain and study an alignment of the linear sequences. The sequence was rotated to start at the single NcoI site, so that position 5759 becomes the new position 1.

The sequence for pIJ86 (map in Figure S1) obtained from JIC StrepStrains ([jic.strepstrains@jic.ac.uk](mailto:jic.strepstrains@jic.ac.uk), John Innes Centre, Norwich Research Park, Norwich, NR4 7UH, United Kingdom) contains the replication protein encoding gene *rep* in opposite orientation to the pIJ101 and pHZ1358 sequence. Thus, the reverse-complementary sequence was analyzed.

The linearized sequences of pIJ101, pHZ1358, and pIJ86 were aligned in pairs with Clone Manager 11, for local alignment (regions of local similarity) with the Needleman-Wunsch algorithm for most optimal alignment, with parameters Match score=1, Mismatch penalty=5, Gap penalty=5 (both changed from the default value of 1, to avoid extension of matching region beyond the high similarity sequence). The results revealed both, a deletion in pHZ1358 (as reported in (Sun *et al.*, 2009)) and that pIJ86 carries a smaller region from the pIJ101 replication region, which does not include the *sti* locus (Figures S4 and S5).

pDS0007 was sequenced with primers JP719, JP720, JP721, JP722, JP723, JP724, JP725, JP726, JP727, JP728, JP729, JP730, JP731, M13rev, pIJ10257_Nde1, pIJ10257_Hind1 (Table S1). Reads from successful sequencing reactions were automatically assembled with GAP4 of Staden Package (Staden *et al.*, 1999; Bonfield and Whitwham, 2010) with default parameters. Afterwards, the *in-silico* compiled sequence for pDS0007 (linearized at the restored HindIII site) was added as reference and the all Sanger reads could be matched to the *in-silico* sequence. Then, linearized sequences of pIJ101 (at position 7209) and pHZ1358 (at position 4336), and pIJ86 (at the unique HindIII site) were added and manually aligned to identify the regions of each vector as present in pHZ1358 and pIJ101. Figure S6 shows the arrangement of all Sanger reads and vector sequences.

**pIJ86**

**5788 bps**

1000

2000

3000

4000

5000

XbaI 265

Acc65I 292

KpnI 292

BamHI 562

XbaI 568

SphI 582

HindIII 588

BglII 600

NcoI 1669

BbsI 1723

FspI 2052

XhoI 2537

EcoRV 2934

MluI 4711

XhoI 4724

NruI 5296

oriT RK2

ermE*p*

ori pIJ101

*pIJ101 rep*

pUC18

ColE1 origin

*aac(3)IV*

Figure S1. Genetic map of pIJ86

Genetic map of pIJ86 based on the *in-silico* sequence provided by JIC StrepStrains. Note the BamHI site located inside the gene *rep* encoding the replication protein in pIJ101 (Fig. S1) does not exist in pIJ86, making the BamHI site at position 562 a unique site for cloning.

Figure S2. Genetic map of pHZ1358

**pHZ1358_AY667410**

**10848 bps**

2000

4000

6000

8000

10000

SpeI 4299

XhoI 5234

BamHI 10848

T7

*bla*

ColE1

repeat_region

*neo*

pIJ101_sti

*pIJ101*_*rep*

*tsr*

repeat_region

*traJ*

bacteriophage lambda COS site

T3

Genetic map of pHZ1358 based on the published sequence GenBank AY667410. Note the BamHI site located inside the gene *rep* encoding the replication protein in pIJ101 (Fig. S3) does not exist in pHZ1358, making the BamHI site at position 10848 a unique site for cloning.

**pIJ101_M21778**

**8830 bps**

2000

4000

6000

8000

BamHI 1

XhoI 42

FspI 527

NcoI 910

NcoI 2038

FspI 2495

NcoI 2693

NcoI 3550

FspI 3891

NcoI 4454

NcoI 4598

NcoI 5759

FspI 7237

SpeI 7243

unknown

*kilB*

unknown

*spdB*

*spdA*

unknown

tra

korA

*korA*

unknown

unknown

*rep*

Figure S3. Genetic map of pIJ101

Genetic map of pIJ101 based on the published sequence GenBank M21778. Note the BamHI site at position 1 (highlighted in red) is located inside the gene *rep,* encoding the replication protein, and the NcoI site at position 5759 (highlighted in green) used for rotation and linearization previous the analyses of similarity.

Figure S4. Pairwise alignment of pIJ86, pHZ1358, and pIJ101

Screenshots from the Clone Manager pairwise alignment analysis showing the regions of high similarity. The coloured frame relates to the colour used in Fig. S5 to represent the regions of similarity. This analysis focuses on the regions originating on pIJ101 present in the other two vectors. There are more regions of similarity between pIJ86 and pHZ1358, which are not shown because they are not relevant to this analysis since they originate from functions needed for replication in *E. coli* (pUC18 *ori*) or conjugation (RK2 conjugation origin).

Figure S5. Representation of the linearized molecules of pIJ86, pHZ1358, and pIJ101

pHZ1358

(10848 bps)

2000

4000

6000

8000

10000

BamHI 1

SpeI 4300

XhoI 5235

'T3

T7

*bla*

ColE1

repeat

*neo*

pIJ101_sti

*pIJ101_rep*

*tsr*

repeat

*traJ*

COS

T3'

pIJ86

(5788 bps)

1000

2000

3000

4000

5000

XhoI 1060

EcoRV 2850

XhoI 3247

FspI 3732

BglII 5184

HindIII 5196

SphI 5202

XbaI 5216

BamHI 5222

KpnI 5492

XbaI 5519

*aac(3)IV*

pUC18-ori

*pIJ101 rep*

ori pIJ101

ermE*p*

oriT RK2

2000

4000

6000

8000

BglII 397

SpeI 1485

BamHI 3073

XhoI 3114

unknown'

*tra*

kiorA

unknown

unknown

*rep*

unknown

*kilB*

unknown

*spdB*

*spdA*

'unknown

pIJ101

(8830 bps)

Representation of the linearized molecules of pIJ86, pHZ1358, and pIJ101 as used for the similarity analysis, highlighting the stretches of molecule of high similarity indicating the sequence from pIJ101 present in pHZ1358 and pIJ86 (100% identity or nearly; regions boxed with same colour; note that the segments span the same length, but the scale of each DNA molecule is different).

Figure S6. Representation of the GAP4 alignment

Representation of the GAP4 alignment (full view on top, zoomed-in at pIJ101-*rep* at the bottom) of vector sequences pDS0007 (top, thicker line), pIJ86 (second from top, as linearized at HindIII site), pIJ101 (third from top, M21778 linearized at position 7209), pHZ1358 (AY667410 linearized at position 4336) and Sanger reads (bottom short arrows). The gene *rep* encoding pIJ101 replication protein is marked in green. The replication region of pIJ101 present in pDS0007 (and therefore also in pIJ86) ends at position 2338 (red vertical line), therefore missing the *sti* region (purple block at the top right of both pIJ101 and pHZ1358 lines). The blue block on the pHZ1358 line represents the 694 bp deletion previously reported (Sun *et al.*, 2009). The yellow block on pIJ101 *rep* gene represents the BamHI site missing in pH1358, pIJ86, and pDS0007.

Figure S7. Analysis of BamHI-free *rep* of pDS0007

Nucleotide alignment of assembled Sanger reads and vector sequences. The numbering of nucleotides assumes the start of pDS0007 as position 1). Top, start of *rep* (green shading) and the three single nucleotide differences (positions 754, 778, and 782) between the published pIJ101 sequence, matching the *in-silico* pIJ86, and published pHZ138 that matches the *de-novo* sequence of pDS0007. Middle, end of *rep* and of the pIJ101 replication region present in pDS0007 (and pIJ86). Bottom, the BamHI site present in pIJ101 but missing in all others is confirmed by electropherograms.

Figure S8. Assessment of the *rep* region from pIJ86 sequence

pIJ101 *rep* contains a BamHI site that is not present in pHZ1358 or pIJ86, but the sequence in the last case was not known. Here, it is confirmed that pIJ86 sequence is the same as pHZ1358, the previously observed mutation inside pIJ101 *rep* 2582C>T that results in the loss of the BamHI site (GGAT**C**C>GGAT**T**C)is confirmed in the original pIJ86 vector, as it was found during the assessment of pDS0007 (Figure S7)

Figure S9. Comparison of new and *in-silico* pIJ86 sequences

Alignment of Sanger sequencing electropherograms showing the differences found between the pIJ86 sequence reported here and the original *in-silico* pIJ86 sequence provided by JIC StrepStrains:

1341G>C; 1365C>G; 1369G>C; 2579N>G; 2580N>A; 2581N>T; 2582N>T; 5296T>C

Figure S10. Alignment of the pIJ86 sequence reported in this study with JIC’s *in-silico* sequence

Alignment of the published pIJ86 sequence (GenBank accession PQ361717) and the original *in-silico* sequence from JIC StrepStrains after rotation to match the start position with the KpnI site (continues on next page).

SFM/Apra plate with replicated exconjugants

SFM/Apra plate with one exconjugant (selection to maintain vector) (**SP+**)

SFM no-antibiotic plate with one exconjugant (no selection pressure to maintain vector) (**SP-**)

Spore stock with selection for vector (**SP+**)

Spore stock without selection for vector (**SP-**)

SFM/Apra

(Test Plate **TP+**)

SFM no-antibiotic (Test Plate **TP-**)

Expected a much lower number of colonies than without Apra, since lack of selection for vector maintenance would have allowed its loss during development.

SFM no-antibiotic (Test Plate **TP-**)

Expected a similar number of colonies as without Apra, since maintenance of the vector was selected during development.

SFM/Apra

(Test Plate **TP+**)

Figure S11. Study on segregational stability

Schematic representation of the study on segregational stability of pGM1190 and pDS0007. The same volume of spore stock dilution was plated on SFM with or without apramycin (Apra). The ratio given is the percentage of colonies on plates with apramycin with respect to the number of colonies appeared without apramycin, i.e. the percentage of colonies that have maintained the plasmid after one sporulation round with or without antibiotic selection. **SP**, Sporulation Plate; **TP**, Test Plate; **+**, supplemented with apramycin; **-**, without apramycin supplementation.

Figure S12. Test of pDS0007 segregation stability

SFM plates supplemented with X-Gluc were inoculated with spores of *S. coelicolor* M145 with pDS0007 obtained from SFM supplemented with apramycin (selection for pDS0007 maintenance, left plate) or without selection (right plate). Most of the colonies (92%) from spores obtained with antibiotic selection show β-glucuronidase activity (blue pigment) indicative of the presence of the vector, while none of the colonies from the spores obtained without antibiotic pressure show any β-glucuronidase activity. This shows that the vector is completely lost without antibiotic selection, and it is lost at low frequency (8%) even with antibiotic selection. Note that the slight purple diffusible pigment observed on the right plate originates from actinorhodin production and not from X-Gluc enzymatic reaction.

**A2**

**B2**

**C2**

**D2**

**C1**

**D1**

**B1**

**A1**

Figure S13. Comparative segregation stability test of pGM1190, and pDS0007

Comparative segregation stability test of pGM1190 (**A** and **B**) and pDS0007 (**C** and **D**) in *S. coelicolor* M145.

Top row (plates numbered with 1) are SFM plates without antibiotic (supplemented with X-Gluc in C1 and D1). Bottom row (plates numbered with 2) are SFM plates supplemented with apramycin to which the colonies of the top plates were replica-plated.

**A1**, spores of *S. coelicolor* M145 carrying pGM1190 obtained under selection with apramycin (SP+ in Figure S11, selection for plasmid maintenance)

**B1**, spores of *S. coelicolor* M145 carrying pGM1190 obtained without selection with apramycin (SP- in Figure S11)

**C1**, spores of *S. coelicolor* M145 carrying pDS0007 obtained under selection with apramycin (SP+ in Figure S11, selection for plasmid maintenance)

**D1**, spores of *S. coelicolor* M145 carrying pDS0007 obtained without selection with apramycin (SP- in Figure S11)

C1 and D1 plates are the same as shown in Figure S12 but three days older.

Most of the colonies with pGM1190 vector grew upon selection with apramycin, indicating that pGM1190 is a very stable vector even without selection. However, colonies with pDS0007 showed only some minor growth and from few colonies upon replica-plating with antibiotic selection, even when the original spore stock had been obtained with antibiotic selection, what indicates that pDS0007 is readily lost even during the growth and sporulation occurred on the top plates (C1 and D1) before replica-plating to the bottom plates (C2 and D2). Note that the purple diffusible pigment observed on top plates, mostly A1 and B1, originates from actinorhodin production and not from X-Gluc enzymatic transformation (seen on top plate C1)

Single spore clone, carrying the vector, growing on SFM without-antibiotic (**SP-)**

(no selection pressure to maintain vector)

Spore stock without selection for vector

SFM no-antibiotic

+X-Gluc (**TP-**)

SFM+Apra

+X-Gluc (**TP+**)

Figure S14. Segregation stability test of pDS0007 in *S. coelicolor* M512

*S. coelicolor* M512 is a double Δ*actII-ORF4* Δ*redD* null mutant [9] that does not produce actinorhodin or prodigiosin, respectively, as to avoid the problem of the blue-purple pigment production that might be mistaken for X-Gluc enzymatic transformation seen in Figure S12 and S13. The same volume of a spore preparation obtained from a plate without apramycin selection (i.e. an **SP-** plate in Figure S11) was used to inoculate two SFM plates supplemented with either only X-Gluc (**TP-**, left) or X-Gluc and apramycin (**TP+**, right). The plate at the left shows abundant number of colonies but very few show conversion of X-Gluc to blue pigment, indicating the very low proportion of spores that have maintained the plasmid. The plate at the right only allows the growth of spores that maintain the plasmid (apramycin selection) and shows a number of individual colonies nearly all of them showing conversion of X-Gluc to blue pigment (which is a second control assay for plasmid backbone maintenance, although in some colonies is not clearly visible in the image).

**pDS0201**

**10877 bps**

2000

4000

6000

8000

10000

AZ031 5306

AZ074 6494

AZ028 7265

AZ033 7372

AZ066 7971

AZ067 8476

AZ029 8743

AZ075 9148

AZ030 10800

I-SceI_site

*gusA*

ermEp*-RBS

lacO

ColE1_ori

*aac (3) IV*

RP4 ori

*J2Z30_008146*

*J2Z30_008147*

*neo*

*ppd_J2Z30_008149*

*J2Z30*_*008150*

Figure S15. Genetic map of pDS0201

Genetic map of pDS0201 construct, based on pDS0007 and used to replace *pepM* with *neo*. Features belonging to the vector are located on the map line, features belonging to the homologous recombination cassette are located inside the map line and coloured in green. The location of relevant oligonucleotides used for cloning of homologous regions and PCR testing of candidate mutants are given outside the map line (AZ0nn).

**A**

**B**

**WT** 2106 bp

**Δ*pepM*::*neo***

2655 bp

AZ074

AZ075

*147*

*neo*

*ppd*

*150*

AZ074

AZ075

*147*

*pepM*

*ppd*

*150*

M	2	3	4	5	8	9	10	WT	M

Kilobases

3 -

2 -

Kilobases

- 3

- 2

Figure S16. Assessment of *S. iranensis* Δ*pepM::neo* candidate mutants by PCR

Assessment of *S. iranensis* candidate double crossover clones, i.e. kanamycin resistant and vector-free (apramycin sensitive, β-glucuronidase negative) by PCR. Oligonucleotides AZ068 and AZ069 anneal either side of the deleted region and generate a PCR product 550 bp larger if the targeted gene *pepM* has been replaced with the kanamycin resistance gene *neo*. **A**, agarose gel electrophoresis of PCR reactions. The only observable amplicon for clones 2 to 10 corresponds to the expected size for the *S. iranensis* Δ*pepM*::*neo* genotype (2655 bp), while the *S. iranensis* parental control (WT) produced only the amplicon with the expected size for the wild-type genotype (2106 bp). DNA size marker is NEB’s 1 kb ladder (from top, 10, 8, 6, 5, 4, **3**, 2, 1.5, 1, 0.5 kb; thicker band is 3 kb). **B,** scheme of the genetic region for both expected mutant and parental strain. The genes *pepM,* encoding the phosphoenolpyruvate mutase, and *ppd*, encoding the phosphonopyruvate decarboxylase, are shown. The nomenclature used for neighbouring genes corresponds to the last three digits of the locus_tag of the GenBank entry (e.g. “147” corresponds to gene J2Z30_008147 of accession JAGGLR010000028; *pepM* is J2Z30_008148, *ppd* is J2Z30_008149)

| Primer pair | Δ*pepM*::*neo*  [gel line clone 3; clone 9] | WT  [gel line] |
| --- | --- | --- |
| AZ066-AZ067 | 506 bp [1;9] | - [5] |
| AZ074-AZ075 | 2655 bp [2;10] | 2106 bp [6] |
| AZ074-AZ067 | 1983 bp [3;11] | - [7] |
| AZ066-AZ075 | 1178 bp [4;12] | - [8] |
| AZ072-AZ073 | - [13;14] | 900 bp [15] |
| AZ084-AZ085 | 5765 bp [16; 17] | 5216 bp [18] |

*S. iranensis* *ΔpepM* #3

*S. iranensis* WT

*S. iranensis* *ΔpepM* #9

#3	#9	WT

**1**

**2**

**3**

**4**

**5**

**6**

**7**

**8**

**9**

**10**

**11**

**12**

**13**

**14**

**15**

#3	#9	WT

**16**

**17**

**18**

**A**

**B**

**C**

**D**

Figure S17. Extended PCR assessment of candidate mutants

Assessment of *S. iranensis* candidate double crossover clones by PCR with an extended number of primer pairs, including two primers that anneal outside of the regions used for homologous recombination. **A**, table of primer pairs, expected amplicon size, and location of sample in the respective gel. **B**, genetic arrangement of the region surrounding *pepM* and location of annealing sites for oligonucleotides in Figure S15-A. Highlighted in green are the oligonucleotides used for amplification of the homologous regions; in blue, oligonucleotides that anneal within the *neo* marker; in orange, oligonucleotides that anneal outside of the homologous regions (and therefore would not anneal on the construct used for production of the mutant). **C** and **D**, images of gels with the results from the test PCR reactions; DNA size marker is NEB’s 1 kb ladder (from top, 10, 8, 6, 5, 4, **3**, 2, 1.5, 1, 0.5 kb; thicker band is 3 kb).

References

Alting-Mees, M.A. and Short, J.M. (1989) pBluescript II: gene mapping vectors. *Nucleic Acids Res* **17**: 9494–9494.

Bethesda Research Laboratories (1986) BRL pUC Host: E. coli DH5alpha Competent Cells. *Focus* **8**: 9.

Bierman, M., Logan, R., O’Brien, K., Seno, E.T., Nagaraja Rao, R., and Schoner, B.E. (1992) Plasmid cloning vectors for the conjugal transfer of DNA from Escherichia coli to Streptomyces spp. *Gene* **116**: 43–49.

Bonfield, J.K. and Whitwham, A. (2010) Gap5—editing the billion fragment sequence assembly. *Bioinformatics* **26**: 1699–1703.

Floriano, B. and Bibb, M. (1996) afsR is a pleiotropic but conditionally required regulatory gene for antibiotic production in Streptomyces coelicolor A3(2). *Molecular Microbiology* **21**: 385–396.

Grant, S.G., Jessee, J., Bloom, F.R., and Hanahan, D. (1990) Differential plasmid rescue from transgenic mouse DNAs into Escherichia coli methylation-restriction mutants. *PNAS* **87**: 4645–4649.

Hamedi, J., Mohammadipanah, F., Klenk, H.-P., Pötter, G., Schumann, P., Spröer, C., et al. (2010) Streptomyces iranensis sp. nov., isolated from soil. *Int J Syst Evol Microbiol* **60**: 1504–1509.

Kendall, K.J. and Cohen, S.N. (1988) Complete nucleotide sequence of the Streptomyces lividans plasmid pIJ101 and correlation of the sequence with genetic properties. *J Bacteriol* **170**: 4634–4651.

Kieser, T., Bibb, M.J., Buttner, M.J., Chater, K.F., and Hopwood, D.A. (2000) Practical Streptomyces Genetics, Kieser, T., Bibb, M.J., Buttner, M.J., Chater, K.F., and Hopwood, D.A. (eds) John Innes Centre, Norwich Research Park, Colney, Norwich NR4 7UH, England: John Innes Foundation.

Ladwig, N., Franz-Wachtel, M., Hezel, F., Soufi, B., Macek, B., Wohlleben, W., and Muth, G. (2015) Control of Morphological Differentiation of Streptomyces coelicolor A3(2) by Phosphorylation of MreC and PBP2. *PLOS ONE* **10**: e0125425.

MacNeil, D.J., Gewain, K.M., Ruby, C.L., Dezeny, G., Gibbons, P.H., and MacNeil, T. (1992) Analysis of Streptomyces avermitilis genes required for avermectin biosynthesis utilizing a novel integration vector. *Gene* **111**: 61–68.

Menges, R., Muth, G., Wohlleben, W., and Stegmann, E. (2007) The ABC transporter Tba of Amycolatopsis balhimycina is required for efficient export of the glycopeptide antibiotic balhimycin. *Appl Microbiol Biotechnol* **77**: 125–134.

Muth, G. (2018) The pSG5-based thermosensitive vector family for genome editing and gene expression in actinomycetes. *Appl Microbiol Biotechnol* **102**: 9067–9080.

Paget, M.S.B., Chamberlin, L., Atrih, A., Foster, S.J., and Buttner, M.J. (1999) Evidence that the Extracytoplasmic Function Sigma Factor ςE Is Required for Normal Cell Wall Structure in Streptomyces coelicolor A3(2). *J Bacteriol* **181**: 204–211.

Rodríguez-García, A., Santamarta, I., Pérez-Redondo, R., Martín, J.F., and Liras, P. (2006) Characterization of a two-gene operon epeRA involved in multidrug resistance in Streptomyces clavuligerus. *Res Microbiol* **157**: 559–568.

Staden, R., Beal, K.F., and Bonfield, J.K. (1999) The Staden Package, 1998. In *Bioinformatics Methods and Protocols*. Methods in Molecular Biology^TM^. Misener, S. and Krawetz, S.A. (eds). Totowa, NJ: Humana Press, pp. 115–130.

Sun, Y., He, X., Liang, J., Zhou, X., and Deng, Z. (2009) Analysis of functions in plasmid pHZ1358 influencing its genetic and structural stability in Streptomyces lividans 1326. *Appl Microbiol Biotechnol* **82**: 303–310.
